# Prevalence and Risk Factors of Intestinal Parasitism in Rural and Remote West Malaysia

**DOI:** 10.1371/journal.pntd.0000974

**Published:** 2011-03-01

**Authors:** Romano Ngui, Saidon Ishak, Chow Sek Chuen, Rohela Mahmud, Yvonne A. L. Lim

**Affiliations:** 1 Department of Parasitology, Faculty of Medicine, University of Malaya, Kuala Lumpur, Malaysia; 2 Department of Orang Asli Affairs, Gombak Hospital, Gombak, Kuala Lumpur, Malaysia; 3 School of Science, Monash University, Bandar Sunway, Selangor Darul Ehsan, Malaysia; National Institute of Parasitic Diseases, China CDC, China

## Abstract

**Background:**

Intestinal parasitic infections (IPIs) have a worldwide distribution and have been identified as one of the most significant causes of illnesses and diseases among the disadvantaged population. In Malaysia, IPIs still persist in some rural areas, and this study was conducted to determine the current epidemiological status and to identify risk factors associated with IPIs among communities residing in rural and remote areas of West Malaysia.

**Methods/Findings:**

A total of 716 participants from 8 villages were involved, comprising those from 1 to 83 years old, 550 (76.8%) participants aged ≤12 years and 166 (23.2%) aged ≥13 years, and 304 (42.5%) male and 412 (57.5%) female. The overall prevalence of IPIs was high (73.2%). Soil-transmitted helminth (STH) infections (73.2%) were significantly more common compared to protozoa infections (21.4%) (*p*<0.001). The prevalence of IPIs showed an age dependency relationship, with significantly higher rates observed among those aged ≤12 years. Multivariate analysis demonstrated that participants aged ≤12 years (OR = 2.23; 95% CI = 1.45–3.45), low household income (OR = 4.93; 95% CI = 3.15–7.73), using untreated water supply (OR = 2.08; 95% CI = 1.36–3.21), and indiscriminate defecation (OR = 5.01; 95% CI = 3.30–7.62) were identified as significant predictors of IPIs among these communities.

**Conclusion:**

Essentially, these findings highlighted that IPIs are highly prevalent among the poor rural communities in West Malaysia. Poverty and low socioeconomic with poor environmental sanitation were indicated as important predictors of IPIs. Effective poverty reduction programmes, promotion of deworming, and mass campaigns to heighten awareness on health and hygiene are urgently needed to reduce IPIs.

## Author Summary

Intestinal parasitic infections (IPIs) are among the most prevalent human afflictions; these infections still have major impact on the socioeconomic and public health of the bottom billion of the world's poorest people. Although Malaysia has a thriving economy, IPIs are still very much prevalent and causing major health problems among the poor and in deprived communities especially in rural and remote areas. A comprehensive study is paramount to determine the current prevalent and factors closely linked to IPIs so that effective control measures can be instituted. In view of this, we conducted this study to provide detailed data of the existing status of IPIs among 716 participants living in rural and remote areas in Peninsular Malaysia. The establishment of such data is beneficial for the public health service to justify and facilitate the reassessment of control strategies and policies in terms of reducing intestinal parasitism. With effective control measures in place, these communities (especially children) will have a greater opportunity for a better future in terms of health and educational achievement and eventually will be at par socially and economically with urban communities in Malaysia.

## Introduction

Globally, the neglected intestinal parasitic infections (IPIs) such as soil*-*transmitted helminth (STH) and protozoa infections have been recognized as one of the most significant causes of illnesses and diseases especially among disadvantaged communities. With an average prevalence rate of 50% in developed world, and almost 95% in developing countries, it is estimated that IPIs result in 450 million illnesses [1], [2], [3]. These infections are ubiquitous with high prevalence among the poor and socioeconomically deprived communities where overcrowding, poor environmental sanitation, low level of education and lack of access to safe water are prevalent [4], trapping them in a perennial cycle of poverty and destitution [5]. These parasitic diseases contribute to economic instability and social marginalization; and the poor people of under developed nations experience a vicious cycle of under nutrition and repeated infections leading to excess morbidity with children being the worst affected [2], [6].

Of these illnesses, infections by STH have been increasingly recognized as an important public health problem and most prevalent of IPIs [7]. STH infections caused by *Ascaris lumbricoides*, *Trichuris trichiura* and hookworm (*Necator americanus* and *Ancylostoma duodenale*) are most significant in the bottom billion of the world's poorest people (i.e., <US$1.25 per day) [8]. To date, approximately one third of the world's population is infected with at least one species of STH, with *A. lumbricoides* infecting 800 million people, *T. trichiura* 600 million, hookworm 600 million and resulting in up to 135,000 deaths annually [5].

With regards to intestinal protozoan infections, giardiasis caused by *Giardia duodenalis*, is the most predominant protozoa infection with an estimated prevalence rates ranging from 2.0 to 7.0% in developed countries and 20.0 to 30.0% in most developing countries, affecting approximately 200 million people worldwide [9]. Amoebiasis caused by *Entamoeba histolytica* is another important pathogenic protozoa affecting approximately 180 million people, of whom 40,000 to 110,000 succumbed to death annually [10]. The opportunistic protozoa, *Cryptosporidium* sp. has also emerged as an important cause of diarrhoeal illnesses worldwide particularly in young children and immunocompromised patients with a prevalence of 4% in developed countries and three to four times more frequent in developing countries [11].

Since the colonial era (i.e., 1930s) in Malaysia, many surveys and studies have been conducted on IPIs, in particular STH infections as they are deemed to be of great medical importance among Malaysian population. While vector-borne diseases such as malaria and filariasis have declined significantly over the years, IPIs which are closely associated with environmental and personal hygiene practices are still causing major health problems among the poor in rural and remote communities in Malaysia [12]. Within this context, we conducted this study to provide a comprehensive data of the current status of IPIs among rural communities residing in remote areas of West Malaysia. The establishment of such data will be beneficial for the public health service to justify and facilitate the reassessment of control strategies and policies.

## Materials and Methods

### Study areas

**Figure 1:**
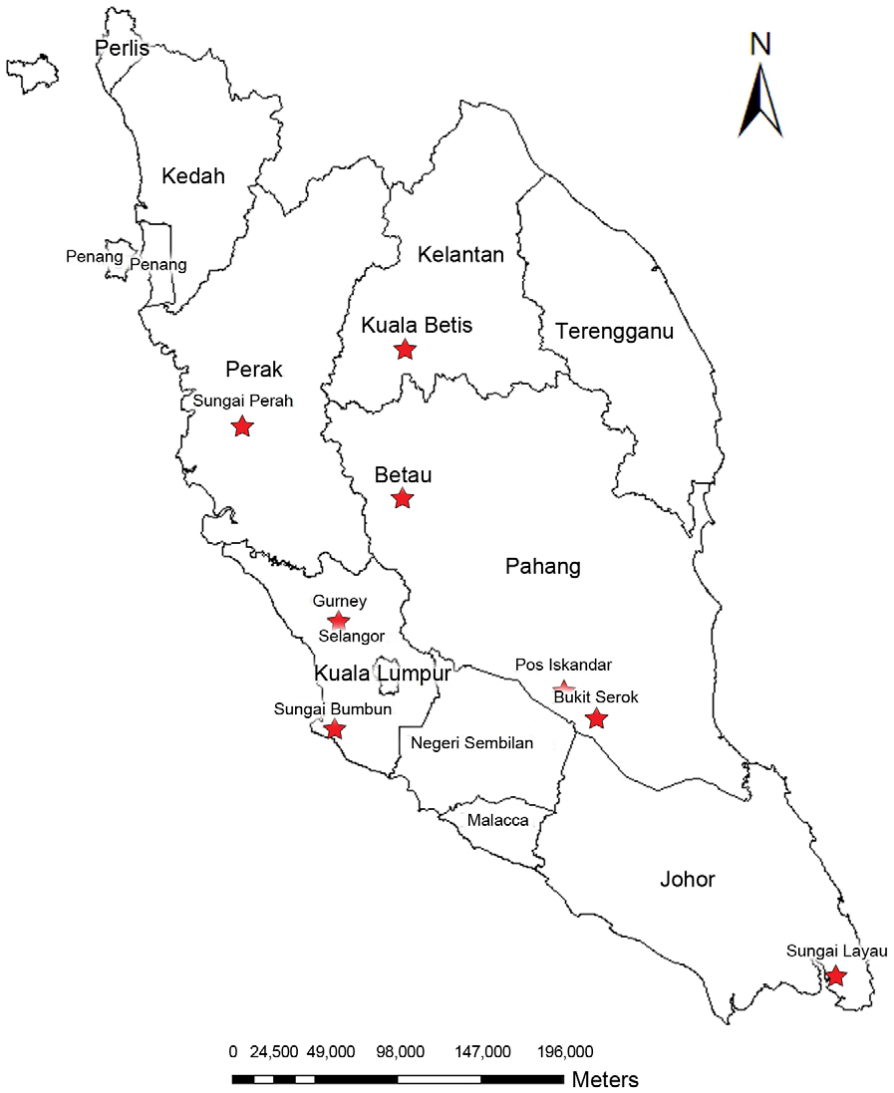
Location of the study areas in West Malaysia (indicated by star symbol).

A cross-sectional study was carried out from November 2007 to July 2009 among 8 villages from 5 different states in rural and remote areas of West Malaysia without being discriminatory towards age or gender. Villages include Betau (101.78°E longitude, 4.10°N latitude), Kuala Betis (101.79°E longitude, 4.90°N latitude), Sungai Bumbun (101.42°E longitude, 2.85°N latitude), Sungai Perah (100.92°E longitude, 4.48°N latitude), Gurney (101.44°E longitude, 3.43°N latitude), Pos Iskandar (102.65°E longitude, 3.06°N latitude), Bukit Serok (102.82°E longitude, 2.91°N latitude) and finally Sungai Layau (104.10°E longitude, 1.53°N latitude) (Figure 1). The villages were selected based on (i) village entry approval by the Ministry of Rural and Regional Development Malaysia and (ii) willingness to participate by the head and community members of the villages.

All villages were located at lowland altitude at the jungle fringes surrounded by rubber and palm oil estates. In general, although these communities have the provision of basic infrastructure (i.e., treated water and electricity) with concrete houses, these facilities are either not fully utilized or evenly distributed. Even if these provisions were given, most of them could not afford to pay their monthly utility bills due to extreme poverty leading to the termination of water and electricity supplies. Therefore, rivers located adjacent to the village remained their main source of water for domestic needs (i.e., drinking, cooking, bathing and washing clothes). Some households still lived in traditional structures of bamboo, wood, brick or a mixture of both with *attap* roof (i.e., thatched roof made from leaves of Nipah palm tree). In addition, although there are pour flush toilets, this facility is not consistently used as the villagers prefer to use nearby bushes and river for defecation. Rearing of animals such as pigs, chickens, ducks, dogs, cats and monkeys are common practices. Most of the residents were employed as unskilled laborers in construction sites, factories, vegetable farms, oil palm and rubber plantations.

### Questionnaire

Before the commencement of the study, an oral briefing explaining the objectives of the study was given to the participants and a voluntary written informed consent was taken from each participant. The participant was then asked by a trained field assistant to answer a pre-tested questionnaire developed to elicit information on the demographic data (i.e., age, gender and education attainment), socioeconomic (i.e., occupation, household income), behavioral (i.e., personal hygiene such as wearing shoes and food consumption), medical treatment (i.e., whether the participant has taken anthelminthic drugs and iron supplement), environmental sanitation and living condition characteristics (i.e., type of water supply, latrine system, garbage disposal system and presence of domestic animals) which will be used to assess the potential risk factors for IPIs. The questionnaire was designed in Bahasa Malaysia, which is the national language for Malaysia and well understood by the participants. For children and very old participants, the questionnaire was completed by interviewing their parents and guardians or the relevant adult (normally head of the family) who signed the informed consent.

### Fecal sample collection and analysis

After consent was obtained and questionnaire answered, a wide mouth screw capped containers pre-labeled with their names and coded were distributed to each participant. Their ability to recognize their names was counter-checked. The participant was instructed to scoop a thumb size fecal sample using a provided scoop into the container, making sure that the sample was not contaminated with urine. Parents and guardians were instructed to monitor their children during the sample collection to ensure that they placed their fecal samples into the right container. Participants who turned up with their fecal samples the following day were honored with a small token of appreciation. The collected fecal samples were processed and examined for the presence of parasites by the formalin ether concentration technique [13]. Briefly, 1 to 2g of fecal sample was mixed with 7 ml of formalin and 3 ml ethyl acetate, centrifuged, stained with 0.85% iodine and examined under light microscope. For *Cryptosporidium* sp., all fecal samples were examined using modified Ziehl Neelsen stain which includes usage of strong carbol fuchsin, 1% acid alcohol and 0.4% malachite green [14]. In addition, Kato-Katz technique was employed to determine the intensity of STH infections, as estimated by egg counts per gram of feces (EPG) as described by Martin and Beaver for *A. lumbricoides*, *T. trichiura* and hookworm [15]. The total number of eggs observed was multiplied with an appropriate exchange number (i.e., number of eggs X 22.2) to give the number of eggs per gram of feces. The worm burden was categorized as light, moderate or heavy intensity based on the threshold proposed by World Health Organization (WHO) Expert Committee in 1987 [16]. Dysenteric or inadequate samples, which were unsuitable for egg counts were used only for examination of intestinal parasites ova by formalin ether concentration technique.

### Statistical analysis

Statistical analysis was carried out using the SPSS software (Statistical Package for the Social Sciences) programme for windows version 13 (SPSS, Chicago, IL, USA). Initial data entry was cross-checked by two independent individuals in order to be sure that data were entered correctly. Before each analysis, data were again checked for consistency. Prevalence of IPIs was determined on the basis of combined results from the different diagnostic methods. For descriptive data, rate (percentage) was used to describe the characteristics of the studied population, including the prevalence of IPIs according to villages, age and gender. The intensity of STH infections (worm burden) was quantitatively estimated as eggs per gram of feces (EPG) and was divided into three main categories: light, moderate or heavy infections and expressed as means. A Pearson's Chi-square (*X^2^*) on proportion was used to test the associations between each variable. In univariate statistical model, the dependent variable was prevalence of IPIs, while the independent variables were sociodemographic, behavior, medical treatment, environmental sanitation and living condition characteristics. All variables that were significantly associated with prevalence of IPIs in univariate model were included in a logistic multivariate analysis using forward elimination model to identify the predictors of IPIs. The level of statistical significance was set as *p*<0.05 and for each statistically significant factor, an odd ratio (OR) and 95% confidence interval (CI) was computed by the univariate and multivariate logistic regression analysis.

### Ethical considerations

The study protocol (Reference Number: 638.36) was approved by the Ethics Committee of the University Malaya Medical Centre (UMMC), Malaysia before the commencement of the study. The participants were informed that the procedure used did not pose any potential risk and their identities and personal particulars will be kept strictly confidential. During the meetings, parents and their children were informed that their participation was voluntarily and they could withdraw from the study at any time without giving any reason. Consent of those who agreed to participate were taken either in written form (signed) or verbally followed by their thumb prints (for those who are illiterate) and from parents or guardians (on behalf of their children).

## Results

### Sociodemographic characteristic

A total of 716 villagers participated in this study. With regards to age groups, there were a total of 550 (76.8%) participants aged ≤12 years and 166 (23.2%) aged ≥13 years ranging from 1 to 83 years with a median age of 11 years and a proportion of 1.1%, 2.4%, 73.3%, 2.0% and 21.2% for the age groups 1 to 4, 5 to 6, 7 to 12, 13 to 17 and above 18 years, respectively. These participants consisted of 304 (42.5%) male and 412 (57.5%) female.

### Prevalence of intestinal parasitic infections (IPIs)

The overall prevalence of IPIs among 716 participants was 73.2% with STH infections (73.2%; 524) being significantly more common compared to protozoa infections (21.4%; 153) (*p*<0.001). In addition, there were also 2 (0.3%) cases of *Fasciolopsis/Fasciola* sp. infection detected (legend indication “other infection”) (Table 1). Prevalence of IPIs were very high in most of the surveyed villages, ranging from 66.7% to 97.8%. Interestingly, infections were very low in Sungai Layau village (4.5%) (Table 1). There was no significant difference of the IPIs between male and female, although female (73.3%) had slightly higher overall prevalence rate compared to male (73.0%) (Table 2). The prevalence of IPIs showed an age dependency relationship, with significantly higher prevalence seen among participants aged ≤12 years compared to those aged ≥13 years (76.7% versus 61.4%, *p*<0.001).With regards to specific age groups, prevalence was highest in the 5 to 6 aged group (94.1%) and lowest (59.2%) among those aged 18 years and above (Table 2).

**Table 1:** Prevalence of intestinal parasitic infections (IPIs) by village and parasite groups (n = 716).

Village name	N	IPIs			
		STH		Protozoa	
		no.	%	no.	%
Betau	92	90	97.8	33	35.9
Kuala Betis	77	72	93.5	30	39.0
Sungai Bumbun	115	103	89.6	25	21.7
Sungai Perah	86	73	84.9	27	31.4
Gurney*	45	37	82.2	7	15.6
Pos Iskandar	113	79	69.9	11	9.7
Bukit Serok	99	66	66.7	16	16.2
Sungai Layau	89	4	4.5	4	4.5
**Total**	**716**	**524**	**73.2**	**153**	**21.4**

N: Number examined; no: Number positive.

* Other infection: There were 2 (0.3%) cases of *Fasciolopsis/Fasciola* sp. infection detected in Gurney village.

**Table 2:** Prevalence of intestinal parasitic infections (IPIs) by gender and age groups (n = 716).

Variables	N	IPIs	
		no.	%
Gender			
Male	304	222	73.0
Female	412	302	73.3
Age groups (years)			
1–4	8	7	85.7
5–6	17	16	94.1
7–12	525	399	76.0
13–17	14	12	85.7
18 and above	152	90	59.2
** Total**	**716**	**524**	**73.2**

N: Number examined; no: Number positive.

### Prevalence and intensity of soil-transmitted helminth (STH) infections

The overall prevalence of STH infections was 73.2% with *T. trichiura* (66.8%) being the most predominant, followed by *A. lumbricoides* (38.5%) while only 12.8% had hookworm infections. In general, participants from Betau village had the highest prevalence of STH infections (97.8%) whilst those from Sungai Layau village (4.5%) had the least. Based on the total sample size (n = 716), double infections (35.6%) were most common, followed by single infections (31.6%) and triple infections (6.0%). *T. trichiura* (28.4%) was the most dominant cause of single infections. The combination of *T. trichiura* and *A. lumbricoides* were the most predominant in the double infections, accounting for 30.6% of the infection rates. With regards to the intensity of infections, all three species of STH showed light to heavy infections. In general, both *T. trichiura* and *A. lumbricoides* infections had similar pattern of worm burden with moderate infection being the most common followed by light and heavy infections. In contrast, most of hookworm infections were light (Table 3).

**Table 3:** Prevalence of soil-transmitted helminth infection according to intensity of infections.

Intensity of infections	Type of infections								
	*T. trichiura* (N = 478)			*A. lumbricoides* (N = 269)			Hookworm (N = 80)		
	no.	%	Mean (EPG)	no.	%	Mean (EPG)	no.	%	Mean (EPG)
Light	186	38.9	498	117	43.5	2,263	60	75.0	41
Moderate	240	50.2	3,249	142	52.8	15,716	19	23.8	185
Heavy	52	10.9	19,716	10	3.7	108,935	1	1.3	NA

N: Number examined; no: Number positive; NA: Not available.

EPG: Eggs per gram.

### Prevalence of protozoa infections

With regards to the protozoa infections, the overall prevalence was 21.4%. The highest prevalence rate was due to *G. duodenalis* (10.4%), followed by *E. histolytica/dispar* (10.2%) and *Cryptosporidium* sp. (2.1%). For protozoa infections, participants from Kuala Betis village (39.0%) had the highest prevalence, whilst those from Sungai Layau village had the least (4.5%) (Table 1). Based on the total sample size (n = 716), single infections (21.4%) were most predominant, followed by double infections (1%). There were no triple infections recorded. As for mix infections with both STH and protozoa, the most common combinations were *T. trichiura*, hookworm and *G. duodenalis* (17.4%) followed by *T. trichiura* either with *G. duodenalis* or *E. histolytica/dispar* (12.4%) and finally a combination of four species which included *T. trichiura, A. lumbricoides, G. duodenalis* and *E. histolytica/dispar* (1.4%) infections.

### Risk factors of intestinal parasitic infections (IPIs)

The risk factors associated with IPIs in relation to sociodemographic and lifestyles among rural communities were examined by univariate analysis. There were eight risk factors identified which included those less than 12 years old (OR = 2.10; 95% CI = 1.43-2.98; *p*<0.001), low household income (OR = 7.60; 95% CI = 5.30–11.13; *p*<0.001), using untreated water supply for daily chores (OR = 2.84; 95% CI = 2.08–3.86; *p*<0.001), the lack of proper latrine system (OR = 2.19; 95% CI = 1.54–3.10; *p*<0.001), non-existence of pour flush toilet (OR = 3.29; 95% CI = 2.62–4.12; *p*<0.001), indiscriminate defecation (OR = 3.45; 95% CI = 2.76–4.32; *p*<0.001) and indiscriminate garbage disposal (OR = 2.06; 95% CI = 1.45–2.94; *p*<0.001), and finally not taking any anthelminthic drugs in the last 12 months (OR = 1.29; 95% CI = 1.01–1.65; *p = *0.038). Although being a female (73.3%; *p* = 0.935), jobless (74.1%; *p* = 0.088), have had no close contact with animals (80%; *p* = 0.193) and not taking any iron supplement (73.5%; *p* = 0.801) were factors which had higher infection rates, nonetheless these variables were not statistically significant (Table 4). Multivariate analysis using forward logistic regression model further confirmed that participant less than 12 years old were 2.2 times (95% CI = 1.45–3.45; *p<*0.001), low household income had 4.9 times (95% CI = 3.15–7.73; *p*<0.001), using untreated water supply had 2.1 times (95% CI = 1.36–3.21; *p<*0.001) and indiscriminate defecation were 5 times (95% CI = 3.30–7.62; *p<*0.001) more likely to suffer from an IPIs, respectively.

**Table 4:** Potential risk factors associated with intestinal parasitic infections (IPIs) (Univariate analysis, n = 716).

Variables	N	IPIs		OR (95%CI)	*p* value
		no.	%		
GenderMaleFemale	304412	222302	73.073.3	0.97 (0.71–1.38)1	0.935
Age (Year)*≤12 years≥13 years	550166	422102	76.761.4	2.10 (1.43–2.98)1	<0.001*
Occupational statusNot workingWorking	64274	47648	74.164.9	1.36 (0.97–1.90)1	0.088
Household income (RM/month)*< RM 500> RM 500	54668	45627	83.540.0	7.60 (5.30–11.13)1	<0.001
Water supply status*Untreated (river, well, rain water)Treated pipe water	317399	275249	86.862.4	2.84 (2.08–3.86)1	<0.001
Presence of proper latrine systemNoYes	212504	181343	85.468.1	2.19 (1.54–3.10)1	<0.001
Type of toilet facilityNonePour flush toilet	538178	44678	82.943.8	3.29 (2.62–4.12)1	<0.001
Defecation places status*Others (Bush, River)Pour flush toilet	550166	45668	82.941.0	3.45 (2.76–4.32)1	<0.001
Close contact with pets/livestockYesNo	65165	47252	72.580.0	0.73 (0.44–1.20)1	0.193
Garbage disposalIndiscriminatelyCollected	198518	168356	84.868.7	2.06 (1.45–2.94)1	<0.001
Iron supplementNoYes	412304	303221	73.572.7	1.03 (0.81–1.32)1	0.801
Anthelminthic drugNoYes	374342	286238	76.569.6	1.29 (1.01-1.65)1	0.038

N: Number examined; no: Number positive.

Reference group marked as OR = 1; CI: Confidence interval.

Significant association (*p*<0.05).

* Variables were confirmed by multivariate analysis as significant predictors of IPIs.

## Discussion

As shown by the results of the present study, intestinal parasitic infections (IPIs) are still a major public health problem (i.e., overall prevalence of 73.2%) among the impoverished and underprivileged communities in rural and remote West Malaysia. However, this study also observed some very encouraging trends. In Sungai Layau village where each family was provided with a concrete house and basic amenities like treated water supply, prevalence of IPIs was shown to be significantly lower (4.5%). This proved that proper provision of basic infrastructure and education are effective tools to reduce the prevalence of these infections. On the contrary, in Betau, Kuala Betis, Sungai Bumbun, Sungai Perah, Gurney, Pos Iskandar and Bukit Serok villages where some villagers still lived in traditional-built houses and using water from wells or rivers, prevalence of IPIs were very high. This was evident in the present findings whereby Betau village which was less provided or developed had the highest rate of infection (97.8%).

Results also showed that STH infections (73.2%) were more common compared to protozoa infections (20.1%). *T. trichiura* infection is the most common (66.8%) followed by *A. lumbricoides* (38.5%) and hookworm (12.8%). These findings were in agreement with other previous local studies where *T. trichiura* infection was found to be the most prevalent (range: 26.0% to 98.2%), followed by *A. lumbricoides* infections (range: 19.0% to 67.8%) and lastly hookworm infections (range: 3.0% to 37.0%) [17], [18], [19], [20], [21]. However, global data has indicated that *A. lumbricoides* infections were the most prevalent among the three STH infections. The higher rate of *T. trichiura* infection has been reported to be due to the ineffective dosage and choice of anthelminthic used. Currently, the recommended treatment regime for STH infection is broad spectrum anthelminthics such as albendazole and mebendazole. Important therapeutic differences do exist between these drugs which affect their uses in clinical practice [22]. Both drugs are effective against ascariasis in single dose, whereas single doses of either albendazole or mebendazole have been found to be ineffective in many cases of trichuriasis [22]. Furthermore, potential resistance of *T. trichiura* to anthelminthic drugs has been highlighted in two intervention studies in Malaysian communities [23], [24].

It has been noted that unscheduled deworming without proper monitoring system was common among the children of these communities. Since the mass deworming program of schoolchildren has been discontinued in 1983 [25], some of the children received anthelminthic drug during visits to health clinic or from their school medical health team. Some parents have also bought anthelminthic drug for their children without following the recommended treatment intervals (i.e., periodic deworming) and this could have resulted in the inefficacy of the drug and subsequently lead to drug resistance [24]. Another important problem encountered in treatment is the high rate of re-infection especially in highly endemic areas. Local studies among rural communities have found that re-infection can occur as early as 2 months post treatment, by 4 months almost half of the treated population had been re-infected [24] and by 6 months the intensity of infections had returned to pre-treatment levels [26]. Similar findings have also been reported in other parts of the world indicating that by 6 months, the intensity of infections of *T. trichiura* and *A. lumbricoides* were similar to pre-treatment levels [27]. WHO has recommended that mass deworming programme should be carried out in communities when the cumulative STH prevalence is more than 50% or the cumulative percentage of moderately or heavily infected individuals is more than 10% [28]. As the present findings have indicated that the overall prevalence was 73.2%, it is strongly recommended that mass deworming programmes are restored and a systematic evaluation of treatment regime must be put in place to reduce the rates of re-infection.

As for protozoa infections, the overall prevalence was 21.4%. However, in contrast with the latest local study in rural area, Noor Azian and colleagues reported very high rates of protozoa infection (72.3%) [29]. The present study found that *G. duodenalis* (10.4%) was the most predominant protozoa, followed by *E. histolytica/dispar* (10.2%) and lastly *Cryptosporidium* sp. (2.1%). In Malaysia, the prevalence of *G. duodenalis* infections varied from 2.0% to 29.2% while the prevalence of *E. histolytica/dispar* infections was reported to range from 1.0% to 18.5% among rural community [23], [29]. Although amoebic liver abscess (65% of 34) has been documented in patients admitted to an urban hospital in Malaysia [30], information from rural communities is not available as this infection can only be confirmed in a hospital setup. Two previous studies have indicated that *Cryptosporidium* sp. infections in rural areas ranged from 5.5% to 20.1% [31], [32].

The present study also reported 2 cases (0.3%) of *Fasciolopsis/Fasciola* sp. infection in Gurney village. This infection is most probably spurious due to consumption of infected animal liver. To date, there has not been any published data on intestinal fluke infection in West Malaysia, however, a case report of fasciolopsiasis by *Fasciolopsis buski* has been reported among rural community in East Malaysia [33]. In addition, two reported cases of food-borne diphyllobothriasis after consuming sushi and sashimi have also been reported in urban West Malaysia [34], [35].

Previous local studies indicated that there was a web of risk factors associated with the high prevalence of IPIs which included age, low family income, inadequate sanitation, presence and close contact with livestock or pets, untreated water supply, low level of parental education, poor geographical and personal hygiene [17], [22], [23]. Using multivariate analysis, the present study confirmed that children, low household income, untreated water supply, indiscriminate defecation were significant risk predictors of IPIs. This finding is further confirmed with the significantly lower prevalence in Sungai Layau village where household incomes are much higher and basic amenities provided by the government are fully utilized by the villagers.

### Conclusion

Intestinal parasitic infections are highly prevalent and are major public health concerns among the poor and socioeconomically deprived rural and remote communities in West Malaysia. Given that IPIs are intimately associated with poverty, poor environmental sanitation and lack of clean water supply, it is crucial that these factors are addressed effectively. Improvement of socioeconomic status, sanitation, health education to promote awareness about health and hygiene together with periodic mass deworming are better strategies to control these infections. With effective control measures in place, these communities (especially children) will have a greater opportunity for a better future in terms of health and educational achievement.

## Supporting Information

Checklist S1 STROBE checklist.
(0.08 MB DOC)
